# Alternatives to Polysomnography for the Diagnosis of Pediatric Obstructive Sleep Apnea

**DOI:** 10.3390/diagnostics13111956

**Published:** 2023-06-03

**Authors:** Taylor B. Teplitzky, Audrey J. Zauher, Amal Isaiah

**Affiliations:** 1 Department of Otorhinolaryngology–Head and Neck Surgery, University of Maryland School of Medicine, Baltimore, MD 21201, USA; 2 Department of Pediatrics, University of Maryland School of Medicine, Baltimore, MD 21201, USA; 3 Department of Diagnostic Radiology and Nuclear Medicine, University of Maryland School of Medicine, Baltimore, MD 21201, USA

**Keywords:** obstructive sleep apnea, sleep disordered breathing, polysomnography

## Abstract

Diagnosis of obstructive sleep apnea (OSA) in children with sleep-disordered breathing (SDB) requires hospital-based, overnight level I polysomnography (PSG). Obtaining a level I PSG can be challenging for children and their caregivers due to the costs, barriers to access, and associated discomfort. Less burdensome methods that approximate pediatric PSG data are needed. The goal of this review is to evaluate and discuss alternatives for evaluating pediatric SDB. To date, wearable devices, single-channel recordings, and home-based PSG have not been validated as suitable replacements for PSG. However, they may play a role in risk stratification or as screening tools for pediatric OSA. Further studies are needed to determine if the combined use of these metrics could predict OSA.

## 1. Introduction

Sleep-disordered breathing (SDB) is defined as the disruption of normal respiration and ventilation cycles during sleep [1]. In children, these disruptions can range from mild snoring to obstructive sleep apnea (OSA). Pediatric OSA affects an estimated 1.2–5.7% of non-obese children [2,3,4] and almost 60% of obese children [5] in the United States. Like childhood obesity [6], the prevalence of pediatric OSA is expected to rise. Besides obesity [7], other risk factors for pediatric OSA include adenotonsillar hypertrophy [8], craniofacial anomalies [9], and neuromuscular disorders [10,11].

In children, OSA can negatively impact quality of life if left untreated [12]. Pediatric OSA has been associated with impaired growth and development [13], as well as behavioral and neurocognitive dysfunction [14]. The first-line treatment is adenotonsillectomy (AT), which results in the improvement or resolution of symptoms in most children [10,15,16].

Level I polysomnography (PSG) is an overnight evaluation performed in an accredited sleep laboratory. The session is attended by a sleep technician and includes a minimum of seven parameters: electrooculography (EOG), electroencephalography (EEG), chin electromyography (EMG), airflow, respiratory effort, oxygen saturation, and electrocardiography (ECG) [17]. The severity of OSA is determined by the apnea-hypopnea index (AHI), or the frequency of partial or complete reduction in airflow. Mild, moderate, or severe OSA correspond to AHI thresholds of less than 5, 5–9, and 10 and over, respectively. Pediatric OSA is diagnosed when the PSG reports an AHI ≥ 1 [17].

Level I PSG is currently the only approved method to diagnose pediatric OSA [10,18]. The American Academy of Sleep Medicine (AASM) [19] and the American Academy of Pediatrics (AAP) [10] recommend PSG to screen children with any SDB symptoms. The American Academy of Otolaryngology–Head and Neck Surgery (AAO–HNS) recommends PSG prior to AT in children under 2 years of age or in those with obesity, craniofacial or neuromuscular disorders, Down syndrome, sickle cell disease, or mucopolysaccharidoses [16]. The AAO–HNS also recommends PSG if the need for surgery is uncertain or if the severity of the SDB cannot be explained by a physical exam [16].

While a PSG is routinely recommended preoperatively by the AASM and the AAP, only 10% of children who are scheduled for AT undergo a PSG [20]. This is likely due to the significant barriers to obtaining a PSG. For example, there is limited access to certified sleep laboratories and providers with the technical expertise necessary to diagnose OSA in infants and young children [21]. The PSG itself is burdensome, requiring the use of multiple monitors during sleep in an unfamiliar laboratory environment [21]. As caregivers need to be present throughout the duration of the test, their employment, productivity, and responsibilities to other members of the family may be impacted. Finally, the cost of PSG in the United States ranges from USD 1000 to USD 4000, posing a marked strain on the healthcare system [22,23].

Less cumbersome diagnostic approaches are necessary. We aim to review alternate options to formal PSG, including single-channel recordings, validated questionnaires, home-based OSA testing, sleep sounds, genetics, and imaging.

## 2. Alternative Approaches to Polysomnography—Objective Measures

### 2.1. Single Channel Recordings

Of the nine parameters included in a level I PSG [21,23,24], cardiovascular, pulse oximetry, body position, and respiratory events have each been examined as potential single-channel equivalents to a standard PSG.

#### 2.1.1. Cardiovascular

Full electrocardiogram (ECG) recordings from overnight PSG have a sensitivity of 85% and specificity of 81% to detect OSA in children (ages 1.2–16 years) when using the modified quadratic discriminant analysis classification system described by Shouldice et al. [25]. Due to the impact of intermittent hypoxia on the heart, ECG-measured heart rate variability may stratify chronic upper airway obstruction [23,26,27,28]. Predictable changes in heart rate variability are noted when evaluating children (ages 3–8 years) with confirmed OSA by PSG [28,29]. Though not accepted by itself, the utility of integrating cardiac rhythm analysis and ECG findings with other screening metrics has not been studied. This represents an opportunity for future research.

#### 2.1.2. Pulse Oximetry

The utility of pulse oximetry as a single-channel recording for predicting pediatric OSA has been extensively studied. Brouillette et al. [30] concluded that pulse oximetry can be used for the potential diagnosis of OSA in infants (ages less than 1 year) with SDB and adenotonsillar hypertrophy. The authors found that a positive nocturnal oximetry trend had a 97% positive predictive value for OSA in infants suspected of having OSA [30]. Models created by Garde et al. [31] demonstrated acceptable accuracy, sensitivity, and specificity when using pulse oximetry to screen for OSA at AHI cut-offs of 1, 5, and 10. The authors concluded that pulse oximetry-based screening for OSA at different AHI thresholds may determine referral thresholds for level I PSG [31]. A meta-analysis performed by Wu et al. [32] found that pulse oximetry had the highest overall specificity when compared to the Pediatric Sleep Questionnaire (PSQ) and the OSA-18 Quality of Life Survey (OSA-18). When combined with the PSQ, the authors noted that pulse oximetry can detect OSA in children (ages 2.9–16.7 years) in instances when formal PSG is unavailable [32].

The McGill Oximetry Score (MOS) developed by Nixon et al. [33] could predict OSA severity, enabling clinicians to prioritize treatment for those with more severe SDB. The MOS is based on the number of clusters of desaturation events and the number of times arterial oxygen percent saturation drops below 90%, 85%, and 80% [33]. The scores, which range from 1 to 4, indicate a normal/inconclusive OSA study (MOS = 1), mild OSA (MOS = 2), moderate OSA (MOS = 3), or severe OSA (MOS = 4) [33]. In an evaluation of a MOS-based treatment algorithm, Horwood et al. [34] concluded that the MOS stratifies SDB in resource-limited scenarios.

The ability of the MOS to detect pediatric OSA and serve as a diagnostic alternative to PSG has been challenged. A study of healthy children (ages 4–18 years) concluded that pulse oximetry was insufficient to detect OSA [35]. Additionally, in a study of children (ages 3–15 years) with adenotonsillar hypertrophy, a high rate of OSA was detected in those assigned a MOS of 1 (inconclusive) [36]. However, the diagnostic accuracy of the MOS may improve with serial overnight oximetry readings. Specifically, two nights of pulse oximetry readings can generate a MOS that supports a diagnosis of OSA when the MOS is at least 2.68 [37].

Outside of the MOS system, abnormal pulse oximetry readings alone may have utility for risk stratification. Pavone et al. [38] found that when PSG is not available, abnormal pulse oximetry readings can predict the child’s need for AT. Similarly, Saito et al. [39] concluded that pulse oximetry can determine indications for AT. Hoppenbrouwer et al. [40] examined pulse oximetry variability in children (mean age 8.3 years) over two consecutive nights: one night of hospital-based PSG followed by one night of home oximetry. The authors found no significant variability between the oximetry measurements obtained in the hospital versus the home setting [40].

Pulse oximetry does not replace formal PSG in pediatric OSA diagnosis. However, the literature suggests it may be used to screen for OSA or to stratify children with known OSA to prioritize treatment.

#### 2.1.3. Body Position

Body position is measured during formal PSG using an actigraph. While a wrist-worn device is most often used [21], sensors worn on the hip or pressure sensors placed in the bed are alternatives [22]. In addition to body position, the actigraph also determines total sleep time (TST), wake after sleep onset (WASO), and sleep efficiency [21]. Multiple actigraph models are available, from the sleep-focused Actiwatch-2^®^ (Phillips Respironics, Amsterdam, The Netherlands) and the Motionlogger^®^ Sleep Watch (Ambulatory Monitoring, Ardsley, NY, USA) to the commercially-available Fitbit Ultra^®^ (Fitbit, San Francisco, CA, USA) and UP^®^ (Jawbone, San Francisco, CA, USA) [21].

In children (ages 5–12 years) with suspected OSA, the Actiwatch-2 demonstrated a sensitivity of 88% (i.e., ability to detect sleep) and a specificity of 46% (i.e., ability to detect wake) [41]. The Actiwatch-2 also underestimated TST and sleep efficiency [41]. Studies of other actigraphs in school-age and adolescent children (ages 3–18 years) found similar patterns of poor specificity but accurate and sensitive estimates of TST, WASO, and sleep efficiency [42,43].

Actigraphy may have greater diagnostic utility when combined with oximetry data. Using machine learning to generate comparison models, Bertoni et al. [44] found that, when combined with the oxygen desaturation index, actigraphy can predict severe OSA in children (ages 2–17 years).

The literature suggesting the use of actigraphy in children is mixed, with a paucity of data on young children. However, the technology is safe, easy to use, and commercially available. The ability of actigraphy alone to diagnose OSA is questionable, though it may be more accurate when used in combination with other metrics such as pulse oximetry. The convenience of actigraphy further warrants the exploration of its diagnostic utility for pediatric OSA.

#### 2.1.4. Respiratory Events

During formal PSG, respiratory events are captured by an oronasal thermal airflow sensor or a nasal pressure transducer [21]. These systems are primarily used in laboratory and research settings. The results of this technology in children are mixed. An observational study of 47 children aged 2–14 years found that the nasal pressure transducer detected more apneas and hypopneas than the thermal airflow sensor [45]. A study of 14 infants (ages 0.5–9.4 months) with suspected OSA noted a nasal pressure transducer was more accurate than a thermal airflow sensor at detecting hypopneas versus apneas [46]. In contrast, a more recent study of 172 children under 3 years old concluded that the nasal pressure transducer had limited ability to detect obstructive events [47].

Single-channel respiratory event recordings cannot replace traditional PSG for diagnosing pediatric OSA. The combination of respiratory data with other single-channel recordings has yet to be studied.

### 2.2. Home Sleep Apnea Testing

Home sleep apnea testing (HSAT) is an unattended, home-based sleep study that utilizes portable and wearable monitors with the goal of replicating level I PSG [23]. Pediatric HSAT uses fewer resources and is more cost efficient than level I PSG, with the additional benefits of increased patient comfort and improved accessibility [18,21]. The AASM does not currently support the use of HSAT to diagnose pediatric OSA. Difficulties in feasibility, validity, identifying arousals and hypoventilation, issues with use in young children or children with comorbidities, and differences in body sizes are the cited challenges [18].

However, the feasibility of HSAT in children (under 16 years of age) has been demonstrated in multiple studies [48,49,50]. A recent systematic review and meta-analysis demonstrated that HSAT had a pooled sensitivity of 74% and a pooled specificity of 90% for detecting OSA in children (ages 1–18 years) [51]. In children aged 2–17 years, HSAT and PSG yielded similar AHI and the lowest oxygen saturation measurements in children ≥ 6 years old [52]. Similarly, in a study of children aged 5–18 years, HSAT demonstrated excellent concordance with level I PSG [53]. The authors concluded that HSAT is a feasible and comparable alternative to level I PSG [53]. A Spanish study found that home respiratory polygraphy is a potential reliable alternative to in-laboratory PSG in children aged 2–14 years [54]. Despite these data, HSAT remains unapproved in children.

While the ability of HSAT to diagnose pediatric OSA holds promise, its current use is limited to a screening method, a role in which it has been successful [51]. To limit the unnecessary use of PSG, home testing may serve as an initial assessment before formal testing is considered [55].

### 2.3. Sound Analysis

During a standard PSG, the brain is monitored for progression through sleep stages. Adult literature has demonstrated changes in breathing patterns associated with different sleep stages [56,57,58,59,60,61], prompting the evaluation of sleep sounds in children. Rembold et al. [62] described the pathophysiology of high-frequency inspiratory sounds (HFIS) generated by children (ages 6–12 years) with obstructive sleep-disordered breathing. Children produce such sounds from a narrowed upper airway, which subsequently acts as a resonating chamber. Similarly, HFIS were shown to be a marker of disturbed breathing in children (ages 6–12 years) with adenotonsillar hypertrophy [63]. With this knowledge, the study of sleep sounds in children as a component of OSA diagnosis or screening has been tested. Using tracheal sounds combined with suprasternal pressure to score respiratory events in children (ages 1–16 years), the sensitivity of detecting an apnea was 86%, and hypopnea was 77% [64]. Similarly, the authors noted a 95% sensitivity and 100% specificity for the detection of an obstructive apnea, a 95% sensitivity and 97% specificity for a mixed apnea, and a 91% sensitivity and 97% specificity for a central apnea [64]. The authors concluded that tracheal sounds with suprasternal pressures have the ability to both detect and characterize apneas and hypopneas in children [64]. The Sonomat system (Sonomedical Pty Ltd., Balmain, NSW, Australia) is a non-invasive mattress pad that can be used in the home without requiring any sensors or monitors to be physically attached to the patient [65]. In a prospective and randomized trial, Sonomat was shown to be reliable and accurate in the diagnosis of adult OSA [65]. The Sonomat was then compared to PSG in children (ages 2–17 years) and found to accurately diagnose OSA [66]. A recent study used the Sonomat to assess snoring and stertor in children (ages 0.8–17.7 years) with OSA and found episodes of these airway sounds occurred more frequently than obstructive events and were associated with sleep disruption [67]. The Sonomat was further found to detect complete and partial airway obstruction in children (under 18 years of age) after sleep surgery [68]. The authors noted the persistence of sleep disturbance, via snoring and stertor, despite improvement in the mixed and obstructive apnea-hypopnea index (MAOHI) [68]. The relationship between snoring sound energy (SSE) and OSA severity, as well as changes in SSE after adenotonsillectomy, were studied in children (ages 6–12 years) [69]. In this article, an SSE of 801–1000 Hertz (Hz) was shown to be significantly related to severe OSA and decreased post-operatively. Similarly, the authors noted that a baseline SSE of 801–1000 Hz significantly predicted surgical success [69]. The authors concluded a potential use of SSE in screening OSA as well as predicting outcomes after OSA treatment [69]. Brietzke et al. [70] found utility with acoustical analysis of snoring in children (ages 2–15 years), noting that an increasing snoring index and loudness were associated with increased OSA severity. The authors noted that increased snoring was associated with oxygen desaturations in cases without OSA [70].

However, the data on sleep sounds have been mixed. A multivariate analysis comparing clinical variables, home snoring sound analysis, and home sleep pulse oximetry did not identify snoring sound energy as a predictor for severe OSA in children aged 5–12 years [71]. A meta-analysis on the acoustic analysis of snoring found that, though accurate, it is not a strong method for diagnosing OSA in those less than 18 years of age [72]. Further studies are needed to understand the utility of sleep sounds in pediatric OSA.

### 2.4. Genetics

OSA is known to cluster in families [73,74], and the study of a genetic component in pediatric OSA confirms it is a heritable trait [75]. However, the genetic inheritance of OSA is likely multifactorial [76], making the identification of causative genes more challenging.

Larkin et al. [77] studied single nucleotide polymorphisms and their association with AHI, noting different gene associations in different populations. The authors found a potential role for genes operating through concomitant diseases, such as obesity, that influence OSA phenotypes [77]. An association between craniofacial anomalies and OSA has been described [78], leading to the study of genes related to structural development. In a study of children with Class III malocclusion, silent mutations in PHOX2b, a gene involved in neural crest cell development for facial and skull growth [79], were found in 32% of children with breathing dysfunction but none of the controls [80]. Additionally, studies about the relationship between OSA and obesity have identified several possible genetic interactions. Ye et al. [81] noted increased expression of liver X receptors, which are important in the control of lipid metabolism. Fatty-acid binding protein 4 (FABP4) gene polymorphisms led to increased morning FABP4 levels in children (ages 2–12 years) with obesity and OSA [82]. Interestingly, this protein was elevated in the morning serum in children (ages 2–12 years) with OSA without obesity [82], indicating these SNPs may portend cardiometabolic risk. Apolipoprotein E (APOE) is also involved in lipid metabolism and has been studied in pediatric OSA. The APOE 4 allele was specifically more frequently identified in children (ages 5–7 years) with OSA, with a higher incidence in those who have neurocognitive effects [83]. A genome-wide association study in children (ages 4–9 years) with OSA noted altered expression of gene clusters involved in inflammation in circulating leukocytes, implying adaptive and end-organ injury in the setting of OSA [84]. Epigenetic studies have shown gene modifications related to pediatric OSA [85], broadening the genetic study to include methylation patterns. The literature supports a genetic influence on metabolic pathways as well as the structural anatomy involved in pediatric OSA, though further research is needed to characterize these relationships.

### 2.5. Imaging

Causes of pediatric OSA can vary, but upper airway narrowing is a universal component. Adenotonsillar hypertrophy is a key factor [86] and is often the dominant anatomic finding in children. However, other craniofacial changes may coexist, with or without an associated syndrome or genetic sequence. Therefore, the role of imaging to identify other areas of collapse has been explored [87].

Cephalometry involves standardized views of the lateral head and neck, allowing for visualization of skeletal and soft tissue structures of the upper airway [87]. Using cephalometry, Shintani et al. [88] confirmed a high prevalence of adenotonsillar hypertrophy in children with OSA. In addition, reduced maxillary and mandibular protrusion and differences in hyoid positioning were significantly associated with OSA [88,89]. Ozdemir et al. [90] used cephalometry as a screening tool and found a significant correlation between these data and AHI in children (ages 4–12 years) with OSA. A systematic review and meta-analysis of craniofacial and upper airway morphology identified a significant association between craniofacial disharmony on cephalometric studies and pediatric sleep-disordered breathing in children less than 18 years of age [91]. However, the authors did not support a causal relationship between facial structure and sleep-disordered breathing [91]. When compared to magnetic resonance imaging (MRI), cephalometric analysis was found to be a valid method to measure nasopharyngeal and retropalatal dimensions in children aged 4.8–9.8 years [92]. Limitations to cephalometry include that the images are obtained upright in awake patients, though OSA affects children in the supine position while asleep, potentially reducing their diagnostic properties [87]. Similarly, the quality of these images is predicated on their positioning, which can alter the results. The images are also limited to 2 dimensions, causing a theoretical loss of important information.

Lateral neck X-rays are plain X-rays of the neck that can provide data on upper airway dimensions. Brooks et al. [93] used lateral X-rays to measure the adenoidal-nasopharyngeal (AN) ratio and found a significant relationship between the AN ratio and the duration of apneas in children aged 0.4–11.6 years. Identification of adenoid hypertrophy on lateral neck X-ray is correlated with the degree of OSA in children aged 4–12 years [94]. Xu et al. [95] utilized lateral neck X-rays as part of a clinical evaluation in children aged 4–18 years and found that upper airway narrowing via adenoid hypertrophy was a significant predictor of OSA. The utility of lateral neck X-ray for identifying or confirming tonsillar hypertrophy is limited in children (mean age 6.2 years) [96] and therefore should be reserved for assessment of the adenoid pad.

MRI allows for cross-sectional evaluation of the upper airway and offers the option of 3D reconstruction as well as the possibility of dynamic images with cinematic (cine) MRI [87]. A benefit of MRI over computerized tomography (CT) is the lack of radiation. However, these tests are long and require the patient to be still, a common challenge in young children. To obtain a useful MRI, sedation may be required, which has inherent risks but may also alter the upper airway dimensions relative to natural sleep [87]. In a study of children aged 7–12 years with PSG-diagnosed OSA, MRI found a positive correlation between tonsil size and the size of the soft palate, with an inverse relationship between AHI and the volume of the oropharynx [97]. The smallest region of the upper airway was found to be the retropalatal airway, which was smaller in those with more severe OSA [97]. MRI tests in 40 children (ages 4–14 years) with diagnosed OSA by PSG were retrospectively evaluated for 25 measurements [98]. The authors identified significant differences in both soft tissue and bony anatomy between those with and without OSA. Specifically, soft tissue differences included a smaller upper airway volume, nasopharyngeal airway, oropharyngeal airway, larger soft palate, and larger tonsils and adenoids in those with OSA [98]. Skeletal differences included a lower hyoid position, smaller sella-nasion-supramentale angle, and smaller mandibular volume in children with OSA [98]. Cine MRI is a specialized MRI study first described by Donnelly et al. [99]. It is a dynamic test, affording the benefit of simultaneous assessment of the entire airway during sleep to identify the site of persistent obstructive airway motion [100,101]. Cine MRI does require anesthesia, and protocols have been made to approximate natural sleep [100], as well as the imaging sequences to obtain an adequate test [101]. Due to the constraints of cost, time, and the need for anesthesia, cine MRI is classically reserved for children with persistent OSA after adenotonsillectomy.

CT scans are fast, noninvasive, and typically readily available. Their main drawback is radiation exposure, though protocols have evolved to reduce the dose in children [87]. Three-dimensional models derived from CT scans in children (ages 3–16 years) were found to predict OSA severity better than clinical scores of upper airway patency [102]. A recent study comparing children under 10 years old with or without OSA found morphological data on the imaging, combined with clinical indices such as habitual snoring, mouth breathing, and adenoid faces, had high diagnostic value for OSA [103]. Further studies are needed to assess the role of CT in pediatric OSA diagnosis.

## 3. Alternative Approaches to Polysomnography—Subjective Measures

### 3.1. Questionnaires

#### 3.1.1. OSA-18 Quality of Life Survey

The OSA-18 Quality of Life Survey (OSA-18) [104] is a validated [105] 18-item questionnaire that uses a Likert-like scale to assess the impact of SDB on five subscales: sleep disturbance, physical suffering, emotional distress, daytime problems, and caregiver concerns. Moderate impact on quality of life correlates with a total symptom score of 60–80, while significant impact correlates with a score over 80 [105].

When compared to formal PSG, the performance of the OSA-18 varies by race and SDB severity. In White children, the OSA-18 has a positive predictive value of 100% when a TSS cut-off of ≥60 and an obstructive AHI > 1 are used [106]. In non-White children, the positive predictive value is 94%, and the specificity of the TSS and AHI cut-offs is only 67% [106]. In all children, the sensitivity and negative predictive value were low [106]. Thus, OSA-18 cannot be used to rule out OSA. When used in children with confirmed moderate or severe OSA by PSG, the OSA-18 cannot accurately diagnose OSA [105]. However, a meta-analysis concluded that while it cannot replace PSG for diagnosis, the OSA-18 can be used to screen for pediatric OSA [32].

While the OSA-18 cannot be used for the diagnosis of OSA in children, it may be considered a screening tool and is validated to assess the impact of OSA on quality of life.

#### 3.1.2. Pediatric Sleep Questionnaire

The Pediatric Sleep Questionnaire (PSQ) is a validated caregiver survey that uses a “yes/no/don’t know” format to capture the child’s frequency and quality of snoring, breathing problems, mouth breathing, daytime sleepiness, inattention/hyperactivity, and other symptoms [107].

In one study, the sleep-related breathing disorder (SRBD) scale of the PSQ had a sensitivity of 71–84%, a specificity of 13%, and a low area under the curve of 0.57–0.69 from receiver operating characteristic curve analysis [108]. Together, these suggest poor overall accuracy for OSA diagnosis [108]. Conclusions from meta-analyses are mixed. Canto et al. [109] determined that the PSQ was not sufficient to replace PSG, while Wu et al. [32] reported that the PSQ is sensitive enough to detect pediatric OSA.

In addition to OSA diagnosis, other alternative uses for the PSQ have been explored. The PSQ items related to behavior impairment, quality of life, and sleepiness have been found to predict improvement after AT and, thus, determine treatment response [110]. One study found that a full PSQ may predict OSA-related neurobehavioral morbidity and response to AT as well as, and sometimes better than, formal PSG in children aged 5–12.9 years [111]. A number of meta-analyses have concluded that the PSQ can be used as a potential screening tool for pediatric OSA [32,109].

In addition to assessing SDB-related quality of life, the PSQ may be useful in screening for OSA or monitoring surgical outcomes.

#### 3.1.3. Children’s Sleep Habits Questionnaire

The Children’s Sleep Habits Questionnaire (CSHQ) [110] is a 45-item questionnaire that uses a 3-point scale to assess child- and caregiver-reported frequency of symptoms [112]. Categories within the questionnaire include bedtime resistance, sleep onset delay, sleep duration, sleep anxiety, night wakening, parasomnias, sleep-disordered breathing, and daytime sleepiness [112]. The CSHQ is validated for use in children aged 4–10 years. While the CSHQ can be used to identify children with sleep disturbances needing additional testing, it cannot replace formal PSG for the diagnosis of pediatric OSA [112].

#### 3.1.4. Refined Questionnaire

Isaiah et al. [113] combined PSQ, OSA-18, and the Epworth Sleepiness Scale data collected from children (ages 5–10 years) undergoing AT. After extracting key features and eliminating redundant items, the refined questionnaire demonstrated improved prediction of OSA severity in children with high a pre-test probability of OSA [113]. A validation study in children aged 3–18 years replicated these results [114]. The use of refined questionnaires that combine key features from the existing surveys may provide an alternate means for OSA diagnosis. Though further evaluation is required, this approach is encouraging.

## 4. Discussion

Level I PSG remains the gold standard for the diagnosis of OSA in children. However, there are inherent barriers to its use. Pediatric PSG is expensive, time-consuming, and uncomfortable. Access to properly equipped sleep laboratories and physicians who can adequately interpret the results is also limited. In this review, we examined three categories of alternative diagnostic approaches to PSG: single-channel recordings, questionnaires, HSAT, sound analysis, genetics, and imaging techniques. The findings are summarized in Table 1. To date, none of these alternatives can independently approximate PSG. However, some of these alternatives, namely combined single-channel recordings, refined questionnaires, HSAT, and CT scans, show promise. Further research in these areas may deliver an accessible and accurate alternative to formal PSG.

## Figures and Tables

**Table 1:** Highlights and challenges of alternative tools for evaluation of pediatric obstructive sleep apnea. OSA obstructive sleep apnea; PSG: polysomnography; AASM: American Academy of Sleep Medicine; MRI: magnetic resonance image; CT: computed tomography; AT: adenotonsillectomy.

Alternative Evaluation	Highlights	Challenges
Objective measures		
Electrocardiogram and heart rate variability	Sensitivity 85%, specificity 81% to detect OSA	Data are lacking Data regarding its use in combination is also lacking Not approved by AASM
Pulse oximetry and McGill Oximetry Score	97% positive predictive value for OSA in children suspected of having OSA Can be considered as a screening tool or potentially diagnostic in situations where PSG is not available	Data are mixed and overall lacking Data regarding its use in combination with other metrics are lacking Not approved by AASM
Body position	Commercially available Ease of use/convenience Safety Accurate and sensitivity estimates of TST, WASO, sleep efficiency	Underestimation of some important sleep metrics in children Poor specificity May give more data when combined with other tools
Respiratory monitoring	Can be used in all ages	May have limited ability to detect obstructive events Typically limited to in laboratory or research settings
Home Sleep Apnea Testing	Unattended, home based Fewer resources required More cost efficient Increased patient comfort Improved accessibility Promising alternative via recent data Can currently be used as a screening tool	Not approved by AASM—cited issues: ○ Not feasible ○ No data on validity ○ Difficulty in identifying arousals and hypoventilation events ○ Challenges with use in very young children or children with comorbidities ○ Difference in pediatric body sizes limiting standardized approach/equipment
Sound Analysis	May detect both obstructive and central events May be added to the screening methods given high sensitivity and specificity	No standardized evaluation/methodology Data are mixed
Genetics	Known genetic component to OSA given it clusters in families Ease of objective testing Have associated genetic findings with OSA	Multifactorial inheritance Isolated genes difficult to identify Cost of testing Genes not found to be causative at this time
Imaging	Multiple imaging modalities have been studied Significant associations between imaging findings and OSA have been described ○ Cephalometry ○ Lateral X-ray or adenoid hypertrophy ○ MRI ○ CT	Imaging findings cannot be considered diagnostic or causal Limits to the imaging options Some imaging requires sedation (MRI) Radiation exposure
Subjective measures		
OSA-18 Quality of Life Survey	Validated questionnaire Can be used to screen for pediatric OSA Validated to assess the impact of OSA on quality of life	Performance of OSA-18 compared to PSG varies by race and severity of sleep disordered breathing Sensitivity and negative predictive value when used alone are low
Pediatric Sleep Questionnaire	Validated questionnaire May determine treatment response after AT/monitor surgical outcomes May be used to screen for pediatric OSA	Poor overall accuracy for OSA diagnosis Data in meta-analysis are mixed
Children’ Sleep Habits Questionnaire	Validated questionnaire Can help identify children with sleep disturbance who need PSG	Not able to diagnose pediatric OSA
Refined Questionnaires	Combination of key components of existing questionnaires has improved prediction of OSA severity	Data are lacking

## Data Availability

No new data were created or analyzed in this study. Data sharing is not applicable to this article.

## References

[1] Gipson K., Lu M., Kinane T.B. (2019). Sleep-Disordered Breathing in Children. Pediatr. Rev..

[2] Bixler E.O. (2009). Sleep Disordered Breathing in Children in a General Population Sample: Prevalence and Risk Factors. Sleep.

[3] Li A.M. (2010). Epidemiology of Obstructive Sleep Apnoea Syndrome in Chinese Children: A Two-Phase Community Study. Thorax.

[4] O’Brien L.M., Holbrook C.R., Mervis C.B., Klaus C.J., Bruner J.L., Raffield T.J., Rutherford J., Mehl R.C., Wang M., Tuell A. (2003). Sleep and Neurobehavioral Characteristics of 5- to 7-Year-Old Children with Parentally Reported Symptoms of Attention-Deficit/Hyperactivity Disorder. Pediatrics.

[5] Verhulst S.L., Van Gaal L., De Backer W., Desager K. (2008). The Prevalence, Anatomical Correlates and Treatment of Sleep-Disordered Breathing in Obese Children and Adolescents. Sleep Med. Rev..

[6] Bryan S., Afful J., Carroll M., Te-Ching C., Orlando D., Fink S., Fryar C. (2021). NHSR 158. National Health and Nutrition Examination Survey 2017–March 2020 Pre-Pandemic Data Files.

[7] Mitchell R.B., Kelly J. (2007). Outcome of Adenotonsillectomy for Obstructive Sleep Apnea in Obese and Normal-Weight Children. Otolaryngol. Head Neck Surg..

[8] Marcus C.L., Loughlin G.M. (1996). Obstructive Sleep Apnea in Children. Semin. Pediatr. Neurol..

[9] Cielo C.M., Marcus C.L. (2015). Obstructive Sleep Apnoea in Children with Craniofacial Syndromes. Paediatr. Respir. Rev..

[10] Marcus C.L. (2012). Diagnosis and Management of Childhood Obstructive Sleep Apnea Syndrome. Pediatrics.

[11] Katz S.L. (2009). Assessment of Sleep-Disordered Breathing in Pediatric Neuromuscular Diseases. Pediatrics.

[12] Baldassari C.M., Mitchell R.B., Schubert C., Rudnick E.F. (2008). Pediatric Obstructive Sleep Apnea and Quality of Life: A Meta-Analysis. Otolaryngol. Head Neck Surg..

[13] Nieminen P., Tolonen U., Löppönen H. (2000). Snoring and Obstructive Sleep Apnea in Children: A 6-Month Follow-up Study. Arch. Otolaryngol. Head Neck Surg..

[14] Landau Y.E. (2012). Impaired Behavioral and Neurocognitive Function in Preschool Children with Obstructive Sleep Apnea. Pediatr. Pulmonol..

[15] (1996). Standards and Indications for Cardiopulmonary Sleep Studies in Children. Am. Thorac. Society. Am. J. Respir. Crit. Care Med..

[16] Mitchell R.B., Archer S.M., Ishman S.L. (2019). Clinical Practice Guideline: Tonsillectomy in Children (Update). Otolaryngol. Head Neck Surg..

[17] El Shayeb M., Topfer L.-A., Stafinski T., Pawluk L., Menon D. (2014). Diagnostic Accuracy of Level 3 Portable Sleep Tests versus Level 1 Polysomnography for Sleep-Disordered Breathing: A Systematic Review and Meta-Analysis. CMAJ.

[18] Kirk V. (2017). American Academy of Sleep Medicine Position Paper for the Use of a Home Sleep Apnea Test for the Diagnosis of OSA in Children. J. Clin. Sleep Med..

[19] Aurora R.N. (2011). Practice Parameters for the Respiratory Indications for Polysomnography in Children. Sleep.

[20] Mitchell R.B., Pereira K.D., Friedman N.R. (2006). Sleep-Disordered Breathing in Children: Survey of Current Practice. Laryngoscope.

[21] Bertoni D., Isaiah A. (2019). Towards Patient-Centered Diagnosis of Pediatric Obstructive Sleep Apnea-A Review of Biomedical Engineering Strategies. Expert. Rev. Med. Devices.

[22] Mitchell M., Werkhaven J.A. (2020). Cost-Effectiveness of Polysomnography in the Management of Pediatric Obstructive Sleep Apnea. Int. J. Pediatr. Otorhinolaryngol..

[23] Bertoni D., Isaiah A. (2020). Home-Based Screening for Obstructive Sleep Apnea in Children. US Respir. Pulm. Dis..

[24] Collop N.A. (2011). Obstructive Sleep Apnea Devices for Out-of-Center (OOC) Testing: Technology Evaluation. J. Clin. Sleep Med..

[25] Shouldice R.B., O’Brien L.M., O’Brien C., de Chazal P., Gozal D., Heneghan C. (2004). Detection of Obstructive Sleep Apnea in Pediatric Subjects using Surface Lead Electrocardiogram Features. Sleep.

[26] Teplitzky T.B., Pereira K.D., Isaiah A. (2019). Echocardiographic Screening in Children with Very Severe Obstructive Sleep Apnea. Int. J. Pediatr. Otorhinolaryngol..

[27] Narkiewicz K. (1998). Altered Cardiovascular Variability in Obstructive Sleep Apnea. Circulation.

[28] Baharav A., Kotagal S., Rubin B.K., Pratt J., Akselrod S. (1999). Autonomic Cardiovascular Control in Children with Obstructive Sleep Apnea. Clin. Auton. Res..

[29] Nisbet L.C. (2013). Nocturnal Autonomic Function in Preschool Children with Sleep-Disordered Breathing. Sleep Med..

[30] Brouillette R.T. (2000). Nocturnal Pulse Oximetry as an Abbreviated Testing Modality for Pediatric Obstructive Sleep Apnea. Pediatrics.

[31] Garde A. (2019). Pediatric Pulse Oximetry-Based OSA Screening at Different Thresholds of the Apnea-Hypopnea Index with an Expression of Uncertainty for Inconclusive Classifications. Sleep Med..

[32] Wu C.-R. (2020). Diagnostic Meta-Analysis of the Pediatric Sleep Questionnaire, OSA-18, and Pulse Oximetry in Detecting Pediatric Obstructive Sleep Apnea Syndrome. Sleep Med. Rev..

[33] Nixon G.M. (2004). Planning Adenotonsillectomy in Children With Obstructive Sleep Apnea: The Role of Overnight Oximetry. Pediatrics.

[34] Horwood L., Brouillette R.T., McGregor C.D., Manoukian J.J., Constantin E. (2014). Testing for Pediatric Obstructive Sleep Apnea When Health Care Resources Are Rationed. JAMA Otolaryngol. Head Neck Surg..

[35] Kirk V.G., Bohn S.G., Flemons W.W., Remmers J.E. (2003). Comparison of Home Oximetry Monitoring with Laboratory Polysomnography in Children. Chest.

[36] Chuanprasitkul C., Veeravigrom M., Sunkonkit K., Tansrirattanawong S., Sritippayawan S. (2021). Incidence/Predictors of Pediatric Obstructive Sleep Apnea with Normal Oximetry. Pediatr. Int..

[37] Pavone M. (2013). Night-to-Night Consistency of at-Home Nocturnal Pulse Oximetry Testing for Obstructive Sleep Apnea in Children. Pediatr. Pulmonol..

[38] Pavone M. (2017). At-Home Pulse Oximetry in Children Undergoing Adenotonsillectomy for Obstructive Sleep Apnea. Eur. J. Pediatr..

[39] Saito H., Araki K., Ozawa H., Mizutari K., Inagaki K., Habu N., Yamashita T., Fujii R., Miyazaki S., Ogawa K. (2007). Pulse-Oximetery Is Useful in Determining the Indications for Adeno-Tonsillectomy in Pediatric Sleep-Disordered Breathing. Int. J. Pediatr. Otorhinolaryngol..

[40] Hoppenbrouwer X.L.R., Rollinson A.U., Dunsmuir D., Ansermino J.M., Dumont G., Oude Nijeweme-d’Hollosy W., Veltink P., Garde A. (2021). Night to Night Variability of Pulse Oximetry Features in Children at Home and at the Hospital. Physiol. Meas..

[41] Meltzer L.J. (2016). Validation of Actigraphy in Middle Childhood. Sleep.

[42] Meltzer L.J., Walsh C.M., Traylor J., Westin A.M.L. (2012). Direct Comparison of Two New Actigraphs and Polysomnography in Children and Adolescents. Sleep.

[43] Toon E. (2016). Comparison of Commercial Wrist-Based and Smartphone Accelerometers, Actigraphy, and PSG in a Clinical Cohort of Children and Adolescents. J. Clin. Sleep Med..

[44] Bertoni D., Sterni L.M., Pereira K.D., Das G., Isaiah A. (2020). Predicting Polysomnographic Severity Thresholds in Children Using Machine Learning. Pediatr. Res..

[45] Serebrisky D., Cordero R., Mandeli J., Kattan M., Lamm C. (2002). Assessment of Inspiratory Flow Limitation in Children with Sleep-Disordered Breathing by a Nasal Cannula Pressure Transducer System. Pediatr. Pulmonol..

[46] Trang H., Leske V., Gaultier C. (2002). Use of Nasal Cannula for Detecting Sleep Apneas and Hypopneas in Infants and Children. Am. J. Respir. Crit. Care Med..

[47] Jurado M.J. (2022). Nasal Cannula Use during Polysomnography in Children Aged under Three with Suspected Sleep Apnea. Sleep Med..

[48] Ioan I. (2020). Feasibility of Parent-Attended Ambulatory Polysomnography in Children with Suspected Obstructive Sleep Apnea. J. Clin. Sleep Med..

[49] Brockmann P.E., Perez J.L., Moya A. (2013). Feasibility of Unattended Home Polysomnography in Children with Sleep-Disordered Breathing. Int. J. Pediatr. Otorhinolaryngol..

[50] Marcus C.L., Traylor J., Biggs S.N. (2014). Feasibility of Comprehensive, Unattended Ambulatory Polysomnography in School-Aged Children. J. Clin. Sleep Med..

[51] Gao X., Li Y., Xu W., Han D. (2021). Diagnostic Accuracy of Level IV Portable Sleep Monitors versus Polysomnography for Pediatric Obstructive Sleep Apnea: A Systematic Review and Meta-Analysis. Sleep Med..

[52] Scalzitti N., Hansen S., Maturo S., Lospinoso J., O’Connor P. (2017). Comparison of Home Sleep Apnea Testing versus Laboratory Polysomnography for the Diagnosis of Obstructive Sleep Apnea in Children. Int. J. Pediatr. Otorhinolaryngol..

[53] Withers A. (2022). Comparison of Home Ambulatory Type 2 Polysomnography with a Portable Monitoring Device and In-Laboratory Type 1 Polysomnography for the Diagnosis of Obstructive Sleep Apnea in Children. J. Clin. Sleep Med..

[54] Alonso-Álvarez M.L. (2015). Reliability of Home Respiratory Polygraphy for the Diagnosis of Sleep Apnea in Children. Chest.

[55] Brunetti L. (2001). Prevalence of Obstructive Sleep Apnea Syndrome in a Cohort of 1,207 Children of Southern Italy. Chest.

[56] Dafna E., Tarasiuk A., Zigel Y. (2015). Sleep-Wake Evaluation from Whole-Night Non-Contact Audio Recordings of Breathing Sounds. PLoS ONE.

[57] Dafna E., Tarasiuk A., Zigel Y. (2012). Sleep-Quality Assessment from Full Night Audio Recordings of Sleep Apnea Patients. Annu. Int. Conf. IEEE Eng. Med. Biol. Soc..

[58] Dafna E., Halevi M., Ben Or D., Tarasiuk A., Zigel Y. (2016). Estimation of Macro Sleep Stages from Whole Night Audio Analysis. Annu. Int. Conf. IEEE Eng. Med. Biol. Soc..

[59] Levartovsky A., Dafna E., Zigel Y., Tarasiuk A. (2016). Breathing and Snoring Sound Characteristics during Sleep in Adults. J. Clin. Sleep Med..

[60] Dafna E., Tarasiuk A., Zigel Y. (2018). Sleep Staging Using Nocturnal Sound Analysis. Sci. Rep..

[61] Kalkbrenner C., Brucher R., Kesztyüs T., Eichenlaub M., Rottbauer W., Scharnbeck D. (2019). Automated Sleep Stage Classification Based on Tracheal Body Sound and Actigraphy. Ger. Med. Sci..

[62] Rembold C.M., Suratt P.M. (2005). An Upper Airway Resonator Model of High-Frequency Inspiratory Sounds in Children with Sleep-Disordered Breathing. J. Appl. Physiol. (1985).

[63] Rembold C.M., Suratt P.M. (2004). Children with Obstructive Sleep-Disordered Breathing Generate High-Frequency Inspiratory Sounds during Sleep. Sleep.

[64] Amaddeo A., Sabil A., Arroyo J.O., De Sanctis L., Griffon L., Baffet G., Khirani S., Fauroux B. (2020). Tracheal Sounds for the Scoring of Sleep Respiratory Events in Children. J. Clin. Sleep Med..

[65] Norman M.B., Middleton S., Erskine O., Middleton P.G., Wheatley J.R., Sullivan C.E. (2014). Validation of the Sonomat: A Contactless Monitoring System Used for the Diagnosis of Sleep Disordered Breathing. Sleep.

[66] Norman M.B., Pithers S.M., Teng A.Y., Waters K.A., Sullivan C.E. (2017). Validation of the Sonomat Against PSG and Quantitative Measurement of Partial Upper Airway Obstruction in Children With Sleep-Disordered Breathing. Sleep.

[67] Norman M.B., Harrison H.C., Waters K.A., Sullivan C.E. (2019). Snoring and Stertor Are Associated with More Sleep Disturbance than Apneas and Hypopneas in Pediatric SDB. Sleep Breath..

[68] Norman M.B., Harrison H.C., Sullivan C.E., Milross M.A. (2022). Measurement of Snoring and Stertor Using the Sonomat to Assess Effectiveness of Upper Airway Surgery in Children. J. Clin. Sleep Med..

[69] Lu C.-T., Li H.-Y., Lee G.-S., Huang Y.-S., Huang C.-G., Chen N.-H., Lee L.-A. (2019). Snoring Sound Energy as a Potential Biomarker for Disease Severity and Surgical Response in Childhood Obstructive Sleep Apnoea: A Pilot Study. Clin. Otolaryngol..

[70] Brietzke S.E., Mair E.A. (2007). Acoustical Analysis of Pediatric Snoring: What Can We Learn?. Otolaryngol. Head Neck Surg..

[71] Hsieh H.-S., Kang C.-J., Chuang H.-H., Zhuo M.-Y., Lee G.-S., Huang Y.-S., Chuang L.-P., Kuo T.B.-J., Yang C.C.-H., Lee L.-A. (2021). Screening Severe Obstructive Sleep Apnea in Children with Snoring. Diagnostics.

[72] Jin H., Lee L.-A., Song L., Li Y., Peng J., Zhong N., Li H.-Y., Zhang X. (2015). Acoustic Analysis of Snoring in the Diagnosis of Obstructive Sleep Apnea Syndrome: A Call for More Rigorous Studies. J. Clin. Sleep Med..

[73] Ovchinsky A., Rao M., Lotwin I., Goldstein N.A. (2002). The Familial Aggregation of Pediatric Obstructive Sleep Apnea Syndrome. Arch. Otolaryngol. Head Neck Surg..

[74] Au C.T., Zhang J., Cheung J.Y.F., Chan K.C.C., Wing Y.K., Li A.M. (2019). Familial Aggregation and Heritability of Obstructive Sleep Apnea Using Children Probands. J. Clin. Sleep Med..

[75] Morell-Garcia D., Peña-Zarza J.A., Sanchís P., Piérola J., de la Peña M., Bauça J.M., Toledo-Pons N., Giménez P., Ribot C., Alonso-Fernández A. (2021). Polysomnographic Characteristics of Snoring Children: A Familial Study of Obstructive Sleep Apnea Syndrome. Arch. Bronconeumol. (Engl. Ed.).

[76] Mainieri G., Montini A., Nicotera A., Di Rosa G., Provini F., Loddo G. (2021). The Genetics of Sleep Disorders in Children: A Narrative Review. Brain Sci..

[77] Larkin E.K., Patel S.R., Goodloe R.J., Li Y., Zhu X., Gray-McGuire C., Adams M.D., Redline S. (2010). A Candidate Gene Study of Obstructive Sleep Apnea in European Americans and African Americans. Am. J. Respir. Crit. Care Med..

[78] Zaffanello M., Antoniazzi F., Tenero L., Nosetti L., Piazza M., Piacentini G. (2018). Sleep-Disordered Breathing in Paediatric Setting: Existing and Upcoming of the Genetic Disorders. Ann. Transl. Med..

[79] Pattyn A., Morin X., Cremer H., Goridis C., Brunet J.F. (1999). The Homeobox Gene Phox2b Is Essential for the Development of Autonomic Neural Crest Derivatives. Nature.

[80] Lavezzi A.M., Casale V., Oneda R., Gioventù S., Matturri L., Farronato G. (2013). Obstructive Sleep Apnea Syndrome (OSAS) in Children with Class III Malocclusion: Involvement of the PHOX2B Gene. Sleep Breath..

[81] Ye X.-H., Chen H., Yu Q., Zhu Q.-L. (2017). Liver X Receptor Gene Expression Is Enhanced in Children with Obstructive Sleep Apnea-Hyperpnoea Syndrome and Cyclooxygenase-2 (COX-2) Is Correlated with Severity of Obstructive Sleep Apnea-Hypopnea Syndrome (OSAHS). Med. Sci. Monit..

[82] Bhushan B., Maddalozzo J., Sheldon S.H., Haymond S., Rychlik K., Lales G.C., Billings K.R. (2014). Metabolic Alterations in Children with Obstructive Sleep Apnea. Int. J. Pediatr. Otorhinolaryngol..

[83] Gozal D., Capdevila O.S., Kheirandish-Gozal L., Crabtree V.M. (2007). APOE Epsilon 4 Allele, Cognitive Dysfunction, and Obstructive Sleep Apnea in Children. Neurology.

[84] Khalyfa A., Capdevila O.S., Buazza M.O., Serpero L.D., Kheirandish-Gozal L., Gozal D. (2009). Genome-Wide Gene Expression Profiling in Children with Non-Obese Obstructive Sleep Apnea. Sleep Med..

[85] Perikleous E., Steiropoulos P., Tzouvelekis A., Nena E., Koffa M., Paraskakis E. (2018). DNA Methylation in Pediatric Obstructive Sleep Apnea: An Overview of Preliminary Findings. Front. Pediatr..

[86] Potsic W.P., Wetmore R.F. (1990). Sleep Disorders and Airway Obstruction in Children. Otolaryngol. Clin. North Am..

[87] Slaats M.A., Van Hoorenbeeck K., Van Eyck A., Vos W.G., De Backer J.W., Boudewyns A., De Backer W., Verhulst S.L. (2015). Upper Airway Imaging in Pediatric Obstructive Sleep Apnea Syndrome. Sleep Med. Rev..

[88] Shintani T., Asakura K., Kataura A. (1996). Adenotonsillar Hypertrophy and Skeletal Morphology of Children with Obstructive Sleep Apnea Syndrome. Acta Otolaryngol. Suppl..

[89] Shintani T., Asakura K., Kataura A. (1997). Evaluation of the Role of Adenotonsillar Hypertrophy and Facial Morphology in Children with Obstructive Sleep Apnea. ORL J. Otorhinolaryngol. Relat. Spec..

[90] Ozdemir H., Altin R., Söğüt A., Cinar F., Mahmutyazicioğlu K., Kart L., Uzun L., Davşanci H., Gündoğdu S., Tomaç N. (2004). Craniofacial Differences According to AHI Scores of Children with Obstructive Sleep Apnoea Syndrome: Cephalometric Study in 39 Patients. Pediatr. Radiol..

[91] Katyal V., Pamula Y., Martin A.J., Daynes C.N., Kennedy J.D., Sampson W.J. (2013). Craniofacial and Upper Airway Morphology in Pediatric Sleep-Disordered Breathing: Systematic Review and Meta-Analysis. Am. J. Orthod. Dentofac. Orthop..

[92] Pirilä-Parkkinen K., Löppönen H., Nieminen P., Tolonen U., Pääkkö E., Pirttiniemi P. (2011). Validity of Upper Airway Assessment in Children: A Clinical, Cephalometric, and MRI Study. Angle Orthod..

[93] Brooks L.J., Stephens B.M., Bacevice A.M. (1998). Adenoid Size Is Related to Severity but Not the Number of Episodes of Obstructive Apnea in Children. J. Pediatr..

[94] Jain A., Sahni J.K. (2002). Polysomnographic Studies in Children Undergoing Adenoidectomy and/or Tonsillectomy. J. Laryngol. Otol..

[95] Xu Z., Cheuk D.K.L., Lee S.L. (2006). Clinical Evaluation in Predicting Childhood Obstructive Sleep Apnea. Chest.

[96] Li A.M., Wong E., Kew J., Hui S., Fok T.F. (2002). Use of Tonsil Size in the Evaluation of Obstructive Sleep Apnoea. Arch. Dis. Child..

[97] Fregosi R.F., Quan S.F., Kaemingk K.L., Morgan W.J., Goodwin J.L., Cabrera R., Gmitro A. (2003). Sleep-Disordered Breathing, Pharyngeal Size and Soft Tissue Anatomy in Children. J. Appl. Physiol..

[98] Cappabianca S., Iaselli F., Negro A., Basile A., Reginelli A., Grassi R., Rotondo A. (2013). Magnetic Resonance Imaging in the Evaluation of Anatomical Risk Factors for Pediatric Obstructive Sleep Apnoea-Hypopnoea: A Pilot Study. Int. J. Pediatr. Otorhinolaryngol..

[99] Donnelly L.F., Surdulescu V., Chini B.A., Casper K.A., Poe S.A., Amin R.S. (2003). Upper Airway Motion Depicted at Cine MR Imaging Performed during Sleep: Comparison between Young Patients with and Those without Obstructive Sleep Apnea. Radiology.

[100] Isaiah A., Kiss E., Olomu P., Koral K., Mitchell R.B. (2018). Characterization of Upper Airway Obstruction Using Cine MRI in Children with Residual Obstructive Sleep Apnea after Adenotonsillectomy. Sleep Med..

[101] Fleck R.J., Shott S.R., Mahmoud M., Ishman S.L., Amin R.S., Donnelly L.F. (2018). Magnetic Resonance Imaging of Obstructive Sleep Apnea in Children. Pediatr. Radiol..

[102] Van Holsbeke C., Vos W., Van Hoorenbeeck K., Boudewyns A., Salgado R., Verdonck P.R., Ramet J., De Backer J., De Backer W., Verhulst S.L. (2013). Functional Respiratory Imaging as a Tool to Assess Upper Airway Patency in Children with Obstructive Sleep Apnea. Sleep Med..

[103] Shi Y., Gu M., Zhang X., Wen M., Li R., Wang Y., Li C., Wang X., Yang R., Xiao X. (2023). Diagnostic Value of Upper Airway Morphological Data Based on CT Volume Scanning Combined with Clinical Indexes in Children with Obstructive Sleep Apnea Syndrome. Front. Med..

[104] Franco R.A., Rosenfeld R.M., Rao M. (2000). Quality of Life for Children with Obstructive Sleep Apnea. Otolaryngol. Head Neck Surg..

[105] Constantin E., Tewfik T.L., Brouillette R.T. (2010). Can the OSA-18 Quality-of-Life Questionnaire Detect Obstructive Sleep Apnea in Children?. Pediatrics.

[106] Ishman S.L. (2015). Is the OSA-18 Predictive of Obstructive Sleep Apnea: Comparison to Polysomnography. Laryngoscope.

[107] Chervin R.D., Hedger K., Dillon J.E., Pituch K.J. (2000). Pediatric Sleep Questionnaire (PSQ): Validity and Reliability of Scales for Sleep-Disordered Breathing, Snoring, Sleepiness, and Behavioral Problems. Sleep Med..

[108] Patel A.P., Meghji S., Phillips J.S. (2020). Accuracy of Clinical Scoring Tools for the Diagnosis of Pediatric Obstructive Sleep Apnea. Laryngoscope.

[109] Luca Canto G. (2014). Diagnostic Capability of Questionnaires and Clinical Examinations to Assess Sleep-Disordered Breathing in Children: A Systematic Review and Meta-Analysis. J. Am. Dent. Assoc..

[110] Rosen C.L. (2015). Utility of Symptoms to Predict Treatment Outcomes in Obstructive Sleep Apnea Syndrome. Pediatrics.

[111] Chervin R.D. (2007). Pediatric Sleep Questionnaire: Prediction of Sleep Apnea and Outcomes. Arch. Otolaryngol. Head Neck Surg..

[112] Owens J.A., Spirito A., McGuinn M. (2000). The Children’s Sleep Habits Questionnaire (CSHQ): Psychometric Properties of a Survey Instrument for School-Aged Children. Sleep.

[113] Isaiah A., Shikara M., Pereira K.D., Das G. (2020). Refining Screening Questionnaires for Prediction of Sleep Apnea Severity in Children. Sleep Breath..

[114] Kennedy C.L., Onwumbiko B.E., Blake J., Pereira K.D., Isaiah A. (2022). Prospective Validation of a Brief Questionnaire for Predicting the Severity of Pediatric Obstructive Sleep Apnea. Int. J. Pediatr. Otorhinolaryngol..

